# Granular Foveolae in the Groove of the Sigmoid Sinus: An Anatomical Study

**DOI:** 10.7759/cureus.36678

**Published:** 2023-03-25

**Authors:** Pavan Guduri, Uduak-Obong I. Ekanem, Devendra Shekhawat, Arada Chaiyamoon, Juan J Cardona, Joe Iwanaga, Aaron S Dumont, R. Shane Tubbs

**Affiliations:** 1 Tulane University School of Medicine, New Orleans, USA; 2 Department of Neurosurgery, Tulane Center for Clinical Neurosciences, Tulane University School of Medicine, New Orleans, USA; 3 Department of Anatomy, Faculty of Medicine, Khon Kaen University, Khon Kaen, THA; 4 Department of Neurology, Tulane Center for Clinical Neurosciences, Tulane University School of Medicine, New Orleans, USA; 5 Department of Anatomical Sciences, St. George's University, St. George's, GRD; 6 Department of Structural & Cellular Biology, Tulane University School of Medicine, New Orleans, USA; 7 Department of Neurosurgery and Ochsner Neuroscience Institute, Ochsner Health System, New Orleans, USA

**Keywords:** dural venous sinus, complications, neurosurgery, skull base, anatomy

## Abstract

Background

Granular foveolae in the groove of the sigmoid sinus have rarely been reported in the literature compared to numerous published reports on the granular foveolae near the superior sagittal sinus and its sulcus on the internal aspect of the calvaria. The present study was performed to better elucidate their prevalence and locations.

Materials and methods

One hundred and ten adult dry skulls (220 sides) were analyzed for the presence of granular foveolae within the groove of the sigmoid sinus. The exact position of the foveolae was documented, and the diameter of the granular foveola was measured.

Results

Granular foveolae were found in the groove of the sigmoid sinus on 3.6% of the sides. These were at or within a mean of 1.3 cm inferior to the transverse-sigmoid junction. When a mastoid foramen was noted in the groove, it was always located inferior to the granular foveolae when present. The mean diameters of the granular foveolae of the left groove of the sigmoid sinus were 2.8 mm and 4 mm for the right grooves. The mean depth of the granular foveolae in the left groove of the sigmoid sinus was 2.7 mm and 3.5 mm for the right grooves. Granular foveolae were statistically larger and deeper on the right versus left sides (p<0.05).

Conclusions

Granular foveolae of the groove of the sigmoid sinus were identified most commonly on the right sides and 3.6% on all sides. If identified on medical imaging, these uncommon structures at the skull base should be considered normal anatomical variations.

## Introduction

Of the intracranial dural venous sinuses, the sigmoid sinus is continuous with the transverse sinus once the transverse sinus leaves the attached edge of the tentorium cerebelli. The grooves of the transverse and sigmoid sinuses of the cranium are indentations created by the dural venous sinuses of the same name [1]. The attached edges of the tentorium cerebelli anchor onto the banks of the grooves of the transverse sinus, which are embedded within the occipital bone. The groove of the sigmoid sinus is a continuation of the groove of the transverse sinus, traveling inferiorly in the temporal bone toward the jugular foramen.

Granular foveolae (arachnoid foveolae) are bony depressions or pits near these sulci created by adjacent arachnoid granulations [2]. While these granulations are not present at birth, they are grossly identifiable by around 18 months in humans [2]. These arachnoid granulations of the posterior cranial fossa must be differentiated from meningoceles, cavernous hemangiomas, dural venous sinus thrombosis, and meningiomas [3,4].

The foveolae created by these arachnoid granulations are commonly associated with and seen near the lacunae laterales along the superior sagittal sinus in the frontoparietal region, and these have been studied in the past [2,3]. What is less well-known and documented is the occurrence of these bony granular foveolae adjacent to the grooves of the transverse and sigmoid sinuses. Although it is well known that arachnoid granulations can occasionally be found in the region of the transverse and sigmoid venous sinuses, the presence of the resulting bony granular foveolae in the same area adjacent to the groove for the sigmoid sinus is what this study aims to demonstrate. It is important to clarify the existence of these granular foveolae in this region so as not to confuse these structures with pathology or emissary vein foramina in the same region.

This article was previously posted to the Research Square preprint server on February 17, 2023.

## Materials and methods

One hundred and ten dry skulls (220 sides) from adult human specimens were analyzed for the presence of granular foveolae within the groove of the sigmoid sinus. The specimens were derived from the osteological collection of our university, Tulane School of Medicine, in New Orleans, Louisiana, USA. Therefore, the majority of these specimens were of North American extract. The exact age and gender of the specimens were not known. However, based on morphology, these skulls were from an approximate age range of 40 to 90 years. When magnification was necessary, a surgical microscope was used (OPMI CS NC31, Carl Zeiss, Oberkochen, Germany). The exact position of the granular foveolae within the groove of the sigmoid sinus was documented in a database as well as with photography. The diameter and depth of the granular foveolae were measured using microcalipers (Mitutoyo, Kawasaki, Japan). Morphometric data were compared based on sides. Descriptive and inferential statistics were calculated with statistical significance set at p<0.05 and using Wizard for Mac. As our study used cadavers, our institution does not require Institutional Review Board approval and is, thus, exempt. The authors state that every effort was made to follow all local and international ethical guidelines and laws that pertain to the use of human cadaveric donors in anatomical research [5].

## Results

**Figure 1:**
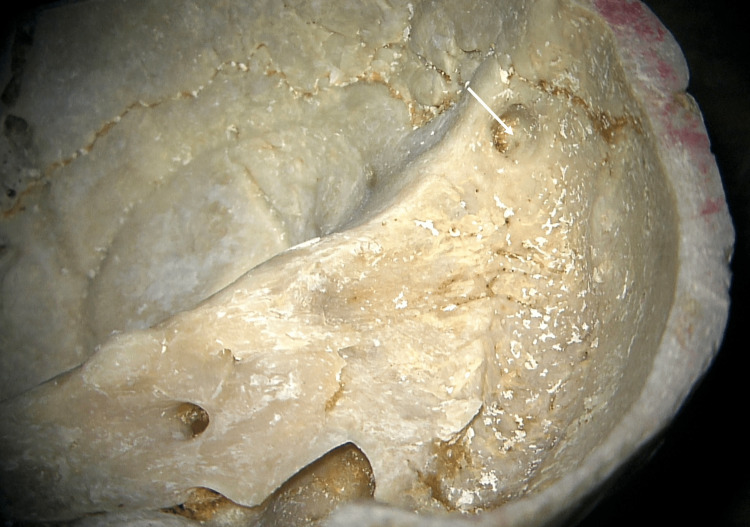
Right-sided groove for the sigmoid sinus with a granular foveola (arrow).

**Figure 2:**
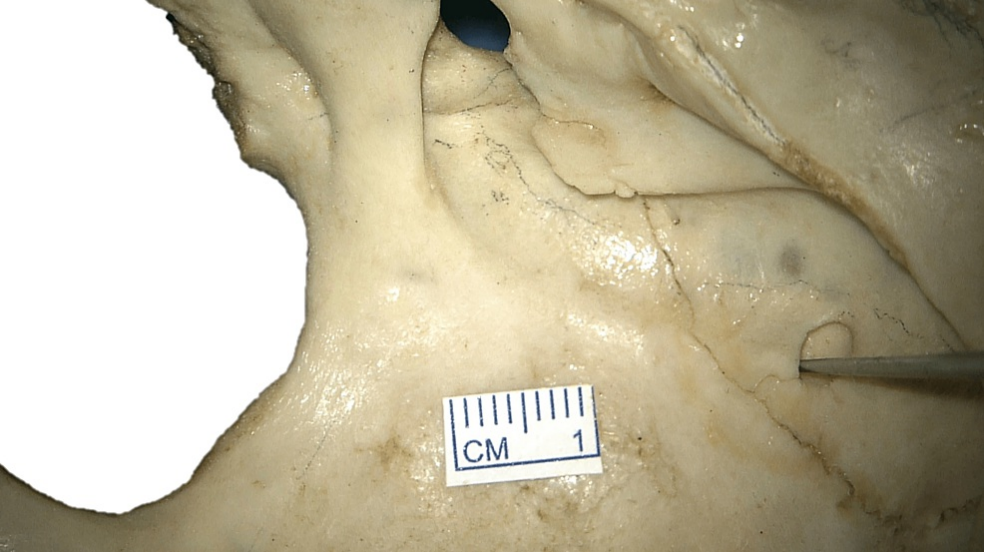
Right-sided granular foveola (probe) just inferior to the transverse-sigmoid junction.

**Figure 3:**
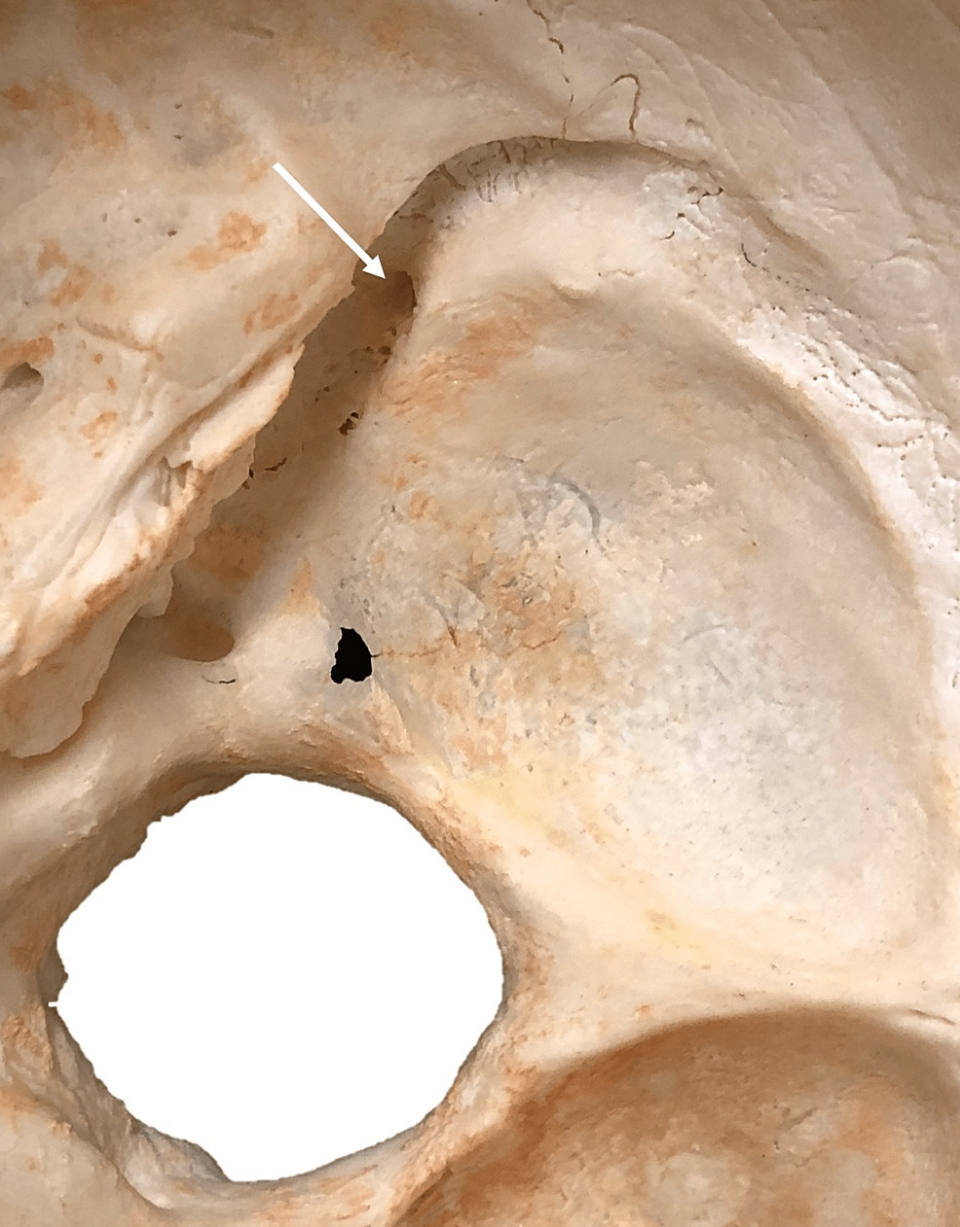
Right-sided granular foveola (arrow) distal to the transverse-sigmoid junction.

Granular foveolae were found in the groove of the sigmoid sinus on eight out of 220 sides (3.6%) (Figures 1-3).

Granular foveola was found on two left sides (0.9%) and six right sides (2.7%). There were no cases of bilaterally located granular foveola. All the granular foveola was located in the groove of the sigmoid sinus and not in the adjacent bone. There was never more than one fovelola in these grooves per side. The granular foveolae of the groove for the sigmoid sinus were most often located in its superior part, near the junction with the groove for the transverse sinus (Figure 1). These granular foveolas were at or within a mean of 1.3 cm (range 0.7-1.46 cm) inferior to the transverse-sigmoid junction. When a mastoid foramen was noted in the groove of the sigmoid sinus, it was always located inferior to the granular foveolae when present. When found together, granular foveolae never communicate with the adjacent mastoid foramen. Granular foveolae of the groove of the sigmoid sinus were most often located on the right sides (n=6; p<0.05). The diameters of the granular foveolae of the left groove of the sigmoid sinus ranged from 2.4-3.3 mm (mean 2.8 mm), and for the right groove of the sigmoid sinus, the diameters of the granular foveolae ranged from 2.5-4.2 mm (mean 4 mm). The depth (Figure 2) of the granular foveolae of the left groove of the sigmoid sinus ranged from 2.5-3.1 mm (mean 2.7 mm), and for the right groove of the sigmoid sinus, the depth of the granular foveolae ranged from 3.1-3.9 mm (mean 3.5 mm). Granular foveolae were statistically larger and deeper for the right versus left sides (p<0.05).

## Discussion

**Figure 4:**
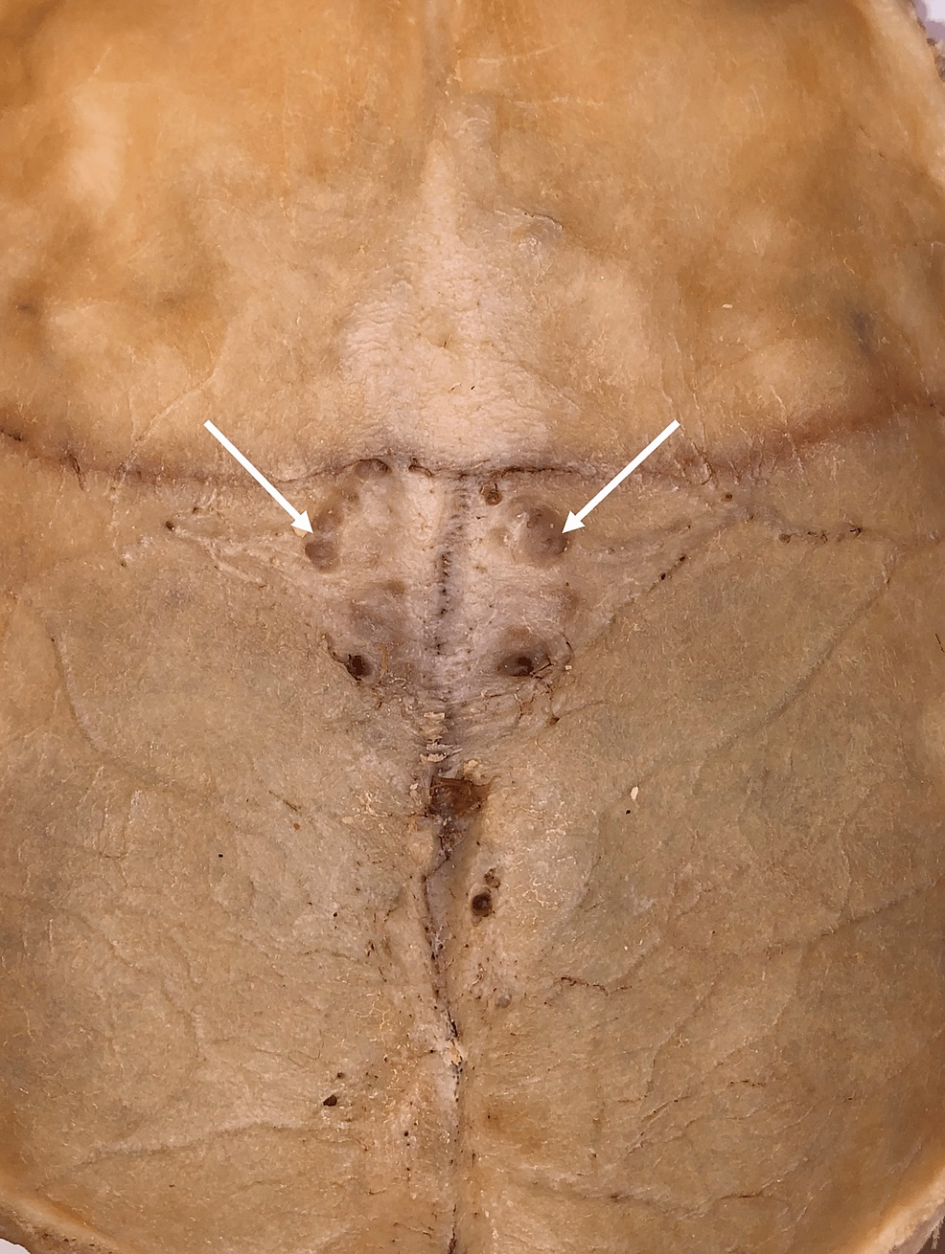
Internal surface of the removed calvaria demonstrating two granular foveola adjacent to the sulcus for the superior sagittal suture.

The foveolae created by the arachnoid granulations, which have been documented in previous studies, are frequently associated with and seen near the lacunae laterales along the superior sagittal sinus (Figure 4).

Herein, we identified granular foveolae in 3.6% of the grooves of the sigmoid sinus, which were statistically more common on the right sides. The existence of granular foveolae adjacent to the groove of the sigmoid sinus is important to highlight, as these foveolae can be mistaken on imaging for pathological structures such as osteolytic lesions, mastoid air cells, thrombus, or emissary vein foramina, e.g., mastoid foramen [6-8]. Branan and Wilson reported in an 18-year-old male the presence of arachnoid granulations that were located in the frontal region bilaterally and simulated multiple osteolytic lesions [8]. Moreover, Sweeney et al. have mapped out these structures along the inner side of the calvaria in order to avoid their injury during craniofacial reconstruction procedures [4].

Tsutsumi et al. [7] evaluated 102 patients with MRI and found arachnoid granulations in the transverse sinus in 41 (40.2% of the right sides and 37.3% of the left sides). Of these, the majority were located in the sinuses' middle and lateral thirds. In the sigmoid sinus, these authors found arachnoid granulations in 18 patients (17.6%) on the right and in 19 patients (18.6%) on the left. In 32 patients, using CT and MRI to evaluate arachnoid granulations in the venous sinuses of the posterior cranial fossa, Roche, and Warner [3] found that the most common location of the arachnoid granulations in the transverse sinus was its middle third, followed by by its lateral third. These authors also found three granulations in the superior portion of the sigmoid sinus and one in its middle portion. Of all granulations, only one showed signs of ‘bone remodeling’ of the inner table of the skull. In addition, they described the presence of the granulations as filling defects seen in MR angiography and digital subtraction angiography, and sometimes they can take an elliptical shape, mainly in oblique views. Such findings are relevant since they can be misleading, and a thrombus could be mistakenly diagnosed. Therefore, these data suggest that granular foveolae are much less common than the number of arachnoid granulations of the posterior cranial fossa in any given patient and present the importance of knowing their features and utilities in clinical scenarios.

Declaration

The authors sincerely thank those who donated their bodies to science so that anatomical research could be performed. Results from such research can potentially increase mankind’s overall knowledge, which can then improve patient care. Therefore, these donors and their families deserve our highest gratitude [9].

## Conclusions

Granular foveolae of the groove of the sigmoid sinus have rarely been reported in the literature compared to numerous published reports on the granular foveolae near the superior sagittal sinus and its sulcus on the internal aspect of the calvaria. These were identified most commonly on the right sides and 3.6% of all sides. If identified on medical imaging, these uncommon structures at the skull base should be considered normal anatomical variations.

## References

[1] Kayalioglu G, Gövsa F, Ertürk M, Arisoy Y, Varol T (1996). An anatomical study of the sigmoid sulcus and related structures. Surg Radiol Anat.

[2] Grossman CB, Potts DG (1974). Arachnoid granulations: radiology and anatomy. Radiology.

[3] Roche J, Warner D (1996). Arachnoid granulations in the transverse and sigmoid sinuses: CT, MR, and MR angiographic appearance of a normal anatomic variation. AJNR Am J Neuroradiol.

[4] Sweeney WM, Afifi AM, Zor F (2011). Anatomic survey of arachnoid foveolae and the clinical correlation to cranial bone grafting. J Craniofac Surg.

[5] Iwanaga J, Singh V, Takeda S (2022). Standardized statement for the ethical use of human cadaveric tissues in anatomy research papers: Recommendations from Anatomical Journal Editors-in-Chief. Clin Anat.

[6] Mayet A, Heil S (1971). Number and distribution of foveolae granulares on the cranial vault of man. Anat Anz.

[7] Tsutsumi S, Ono H, Ishii H (2021). Arachnoid granulations bulging into the transverse sinus, sigmoid sinus, straight sinus, and confluens sinuum: a magnetic resonance imaging study. Surg Radiol Anat.

[8] Branan R, Wilson CB (1976). Arachnoid granulations simulating osteolytic lesions of the calvarium. AJR Am J Roentgenol.

[9] Iwanaga J, Singh V, Ohtsuka A (2021). Acknowledging the use of human cadaveric tissues in research papers: Recommendations from anatomical journal editors. Clin Anat.

